# Awareness and Use of Benzodiazepines in Healthy Volunteers and Ambulatory Patients Visiting a Tertiary Care Hospital: A Cross Sectional Survey

**DOI:** 10.1371/journal.pone.0001804

**Published:** 2008-03-19

**Authors:** Mustafa Raoof, Haq Nawaz, Rabeeya Nusrat, Aqueel Hussain Pabaney, Ali Raza Randhawa, Rabeea Rehman, Nida Butool Rizvi, Haider Naqvi

**Affiliations:** 1 Aga Khan University, Medical College, Karachi, Pakistan; 2 Department of Psychiatry, Aga Khan University, Medical College, Karachi, Pakistan; Cornell University, United States of America

## Abstract

**Background:**

Indiscriminate prescription of Benzodiazepines in Pakistan and subsequent availability over-the-counter without prescription is a major public health problem, requiring systematic inquiry through research. Additionally, there is limited data on the awareness and use of Benzodiazepines from developing countries making it impossible to devise meaningful health policies.

**Methodology/Principal Findings:**

This was an Observational, Cross-Sectional study. conducted at Aga Khan University. A total of 475 (58.5% males, 41.5% females) people visiting a tertiary care hospital were interviewed by means of a structured questionnaire. The results showed that majority of population was aware of one or more Benzodiazepines (80.4%) and 30.4% had used them at some point in life. 42.4% of the users had been using it for more than a year. Commonest reason for use was sleep disturbance. Frequency of usage was higher for females, married individuals, educated (>Grade12), high socioeconomic status and housewives. More (59%) were prescribed than not and of them most by GP (58.5%). Only 36.5% of them were particularly told about the long-term addiction potential by the use of these drugs.

**Conclusion:**

Easy availability, access to re-fills without prescription and self prescription compounded with the lack of understanding of abuse potential of benzodiazepines constitutes a significant problem demanding serious consideration from health policy makers.

## Introduction

Leo Sternbach (1908–2005) is known as the pioneer of benzodiazepine (BDZ) tranquilizers. His discovery of chlordiazepoxide in 1957 lead to the introduction of a large number of similar compounds in clinical practice [1]. Today, benzodiazepines are amongst the most widely used group of drugs worldwide [2].

Benzodiazepines are commonly used for a number of psychiatric and non-psychiatric reasons some of which include relief of anxiety and insomnia, treatment of *delirium tremens* and other withdrawal syndromes, pre-operative sedation, treatment of epileptic fits, and relief of muscle spasticity [2], [3].

The most common adverse effects associated with benzodiazepines are residual sedation, anterograde amnesia, and rebound insomnia [4]. These effects are dose related and can therefore be minimized with dose reduction [5]. Although they are considered safe and are generally well-tolerated, there is a considerable risk of misuse and abuse [2].

The occurrence of dependence and withdrawal symptoms is directly dependant on the duration of use [6]. Most patients with four months or more of therapy with benzodiazepines experience some kind withdrawal symptoms including anxiety, dysphoria, malaise, depersonalization or perceptual changes such as hyperacusis and unsteadiness [7].

BDZ prescription and usage is regulated in most developed countries. This however, does not hold true for developing countries where such agents are available over-the-counter (OTC) [2], [8], [9]. Easy OTC availability and popularity as ‘sleeping pills’ make these agents the preferred drugs for self-poisoning in Pakistan [9].

BDZ are available over the counter with out prescription. Health care delivery system is also in shambles. Majority (almost 80%) seek health care as an out-of-pocket expenditure. Only poorest of the poor seek health care services from public sector hospitals. Private health care, for most part is unregulated. BDZ are dispatched indiscriminately by general physicians–at times with ulterior motives. Therefore addiction and dependence is a major issue. There is no research exploring this issue systematically.

The purpose of this study therefore is to assess the awareness and demographic risk factors associated with BDZ use in Pakistani population. We also estimate the life-time prevalence of BDZ use in this population in order to gauge the magnitude of the problems associated with OTC availability of benzodiazepines.

## Methods

### Study design

This was an observational, cross sectional study. A questionnaire-based survey was conducted at the Aga Khan Hospital over a three-week period in October 2004.

### Sampling Method and Ethical Concerns

The study was based on a sample of convenience. Systematic effort was made to obtain data from all consecutive out-patients and visitors in all major waiting areas of the out-patient department during business hours 9:00am–5:00pm. Three random weekdays were chosen (in a three week period) for this purpose. The number of those who refused to participate, reason for refusal and their demographics were not recorded. Exclusion criteria included age less than 15 years, all inpatients, psychiatry clinic outpatients, medical professionals, health employees and their family members.

Since our study was not experimental and did not involve any intervention, we did not approach an ethics committee for review before conducting the study. We took informed consent from all participants and maintained strict confidentiality. All possible ethical concerns were discussed with the supervising faculty in the Department of Psychiatry at our University. All ethical principles of the Helsinki Declaration were followed.

### Data Collection

There is no research exploring this issue systematically. Thus no questionnaires were available that explore the issue from public health or mental health perspective. Since conditions pertaining to use of BDZ are unique in Pakistan, we decided to design our own instrument. This was done in consultation with the experts in the field of psychiatry and public health. It was designed to assess various aspects of knowledge, practices and attitude towards the use of benzodiazepines. As the questionnaire was self-administered it was translated in the local language (Urdu) and piloted. The interviewers were trained in both English and Urdu for standardization.

To assess knowledge regarding benzodiazepines, subjects were asked if they recognize the names of some of the commonly prescribed BDZ. Those who had recognized the name of at least one of the commonly prescribed BDZ were further asked if they thought these drugs are harmful. Further questioning regarding practices and attitude was not possible in those who failed to recognize the name of any of the commonly used benzodiazepines

Those who had heard of a BDZ were further divided into two groups depending on usage of the drug (i.e. those who had used benzodiazepines and those who had not used benzodiazepines). All subjects who had used or were currently using these medications were asked about the drug(s) they had used, the reason and duration of use.

In order to assess the prescription practices, subjects were further asked whether the drug(s) had been prescribed or if they had been self medicating, designation of prescribing physician, if they had been informed about the side effects of these drugs or not and finally, if they had been specifically warned about their addiction potential or not.

### Analysis

The data was tabulated and analyzed in SPSS (version 12.0). Descriptive statistics were obtained on responses to each question. Chi square test, Student's t-test and one way ANOVA were used for inferential statistics.

## Results

### Demographics

In this study 475 individuals participated. Mean age of the population was 35.64±11.06 years. 58.5% were males (n = 278) and 41.5% were females (n = 197). Majority were married (73.5%) as opposed to single (26.5%). Most of the subjects were housewives (30.3%) or professionals (20%) among others. 49% of the study population comprised of people from medium income group (Rs 5000–25000/mo), 24% from high income group (>Rs 25000) and 19.6% from low income group (<Rs 5000). Important demographic measures are summarized in Table 1

**Table 1:** Demographic Distribution (n = 475)

Variable	Subcategory	Frequency (%)
Gender	Male	278 (58.5)
	Female	197 (41.5)
Age (years)	1 (16–27)	148 (31.2)
	2 (28–39)	166 (34.9)
	3 (40–51)	102 (21.5)
	4 (52–63)	42 (8.8)
	5 (64–75)	15 (3.2)
Marital status	Married	349 (73.5)
	single	126 (26.5)
Occupation	Housewife	144 (30.3)
	Professionals	95 (20.0)
	Businessmen	66 (13.9)
	Students	57 (12.0)
	Laborers	44 (9.3)
	Unemployed	21 (4.4)
Income per month	Low (<Rs 5000)	93 (19.6)
	Medium (Rs 5000–25000)	233 (49.1)
	High (>Rs 25000)	114 (24.0)
Education	Primary or less	71 (14.9)
	Secondary	165 (34.7)
	Graduate and above	239 (50.3)

### Knowledge

Of the sampled population 80.4% had heard of a BDZ. Proportion of males (84.5%) who had heard of one or more drugs was significantly greater than proportion of females (74.6%) who had heard of one or more drugs (p<0.05). There was a significant association (p = 0.018) between occupation and those who had heard of a BDZ (Professionals 88.4%, Laborers 84.1%, Businessmen 83.3%, house-wives 74.3%, Students 73.7%, Unemployed 66.7%).

The results demonstrated that 86% of the high income (>Rs 25000/mo) group had heard of a BDZ compared with 82% and 65% of the middle (Rs 5000–25000/mo) and low income (<Rs 5000/mo) group respectively (p<0.001). Education was significantly associated (p<0.001) with the awareness of the name of a BDZ (Graduate and above 90.4%; up to secondary 77.6%; primary or less 53.5%). The data however, failed to show an association of age and marital status with the awareness of the name of a BDZ.

Of those who had heard of one or more of the drugs 68.9% thought that they can be harmful, 17.3 % did not think so while 13.8% did not know if they can be harmful or not. There was a significant association between awareness of harm and education (p<0.001) i.e. 77.8% of graduates and above, 63.8% of those educated up to secondary and 35.1% of those educated up to primary or less thought that BDZ can cause harm.

### Attitudes and Practices

In overwhelming majority (58.1%), the source of information regarding benzodiazepines were family and friends. Others learnt about the drugs from general physicians (18.3%), specialist physicians (4.2%) and through media (3.9%) and psychiatrists (1.6%).

The life time prevalence of use was reported to be 30.4% (n = 144). Of them, 33 had used multiple medications. The commonly used medications were Alprazolam(37.9%), Bromazepam(34.5%), Diazepam(10.7%), Lorazepam(8.4%) among others (30.3%). Commonly reported reasons for use were sleeplessness (68.3%), stress or anxiety (52.2%), *ghabrahat–*a cluster of symptoms that includes a vague sense of uneasiness, fidgetiness, restlessness or impending doom (10.9%), and depression (7%). Percentages may not add up to 100% because of multiple responses by each respondent to the questions regarding source of information, name of drug used and reason for use.

In our study mean age of those using BDZ was higher (41.6±13.6 yrs) when compared with the non-users (33±10.5 yrs) (p<0.001). More married individuals were using BDZ then single (34% vs. 20%). Among all the professional groups, homemakers (Housewives) were particularly prone to the use of BDZ (p = 0.001). Income level was also a significant variable (p<0.001) influencing BDZ use; highest in the high income group (42.5%) with decreasing use in middle (27.9%) and low income groups (20.7%). Higher educational status was associated with the use of BDZ (p = 0.003). Inferences regarding usage of benzodiazepines and various demographic variables along with p-values are summarized in Table 2.

**Table 2:** Characteristics of persons who reported BDZ use

Variable	Sub-category	Percentage use in each subcategory	Mean±Std. dev	P-value
Gender	Male	25.3		p<0.05
	Female	37.8		
Age (years)	BDZ users		41.65±13.57	p<0.001*
	BDZ non-users		33.03±10.54	
Marital status	Married	34		p<0.05
	single	20.6		
Occupation	Housewife	39.9		p = 0.001
	Professionals	25.3		
	Students	24.6		
	Businessmen	22.7		
	Laborers	20.5		
	Unemployed	10		
Income per month	Low (<Rs 5000)	20.7		p<0.05
	Medium (Rs 5000–25000)	27.9		
	High (>Rs 25000)	42.5		
Education	Primary or less	15.7		p = 0.003
	Secondary	28		
	Graduate and above	36.4		

* Student's t-test; For the analysis of remaining variables Chi-square test was employed

Most of the users (59%) reported that the drugs were prescribed to them by health care provider at some point in time; 58.8% of them were prescribed by GP, 35.3% by specialists, 5.9% by psychiatrists. Of those who were prescribed, 41.2% were informed about the side effects of these drugs and 36.5% were particularly told about the long-term addiction potential. The study failed to show an association between provision of information and the category of prescribing physician which may be due to lack of power to show a significant association.

The greatest proportion of users had taken the drug for more than one year (42.4%). 23.6% of people had used it for a month or less and 11.8% had used it for more than a month (but less than a year). 22.3% did not remember the exact duration of use.

## Discussion

This is the first study from Pakistan that reports prevalence estimates of BDZ use in a non-admitted population, describing socio-demographic correlates.

The life-time prevalence of BDZ use was 30.4% in our data. This is much greater than that of USA (4.1%)[10]. The life-time prevalence in a multi-center study across Brazil was reported to be 3.3%[11]. Our data is comparable to life-time prevalence reported from Chile (28.4%)[12].

BDZ usage was more common in females (37.8% vs. 25.3% in males) and the users tended to be older. This is consistent with studies from developed countries [13], [14], [15], [16]. When data (not shown) was stratified to elderly (≥60 years) and non-elderly (<60 years), we failed to show an association between these sub-groups and BDZ usage. This may be explained by the small percentage of elderly in our sample and a subsequent lack of power to show a significant association. BDZ use was higher in married people as compared to single in our cohort. This is contrary to the finding reported by Swartz et al. who found that BDZ use was higher in separated or divorced North Americans [14]. Our findings are consistent with earlier studies that have reported marriage as a psychosocial risk factor for mental illnesses in Pakistan [17].

We observed that people who had high income and higher education comprised a greater proportion of BDZ users. Swartz et al. reported higher use in less educated whereas Neutel et al. have reported higher use in those North Americans who have completed high school [14], [16]. Approximately 40% of the house wives used BDZ at some point in their life. This may be explained by studies that have reported marriage as a psychosocial correlate for mental illness in Pakistan as discussed above. Only 10% of unemployed men had used BDZ which is contrary to the higher rate reported by Parma et al [13].

Contrary to the prescription practices in developed countries, we observed that only 59% of BDZ users were prescribed these agents at some point [2]. Only 36.5% of these, however, were informed about the long term abuse potential of benzodiazepines. This fact underscores the importance of physicians educating their patients about the long term abuse potential of benzodiazepines especially where patients have easy OTC access to these agents. Long term use (>1 year) was seen in 42.4% of BDZ users in our study. Magrini et al. and Lyndon et al. have described long-term use (>6 months) in 56% and 82% of Italian and rural Australian users respectively [13], [15]. Neutel et al. observed that 53.2% of BDZ users continue using these agents for at least 2 years [18]. Commonest reason for use was sleeplessness/insomnia closely followed by anxiety. This is consistent with the European data reported by Lader et al [19].

It was interesting to note that, a significantly greater proportion of non-users (61%) as opposed to users (39%) thought that these drugs may cause some kind of harm (p<0.001). This highlights the importance of awareness programs and primary prevention. However, among those who particularly knew that these drugs can be abused, 68.7 % were users. Long term users of BDZ most likely have had the opportunity to acquire specific knowledge of abuse, side effects and brand names. It is also possible that they need to use these medications for clinical reasons despite the awareness of abuse potential.

The findings of this study need to be interpreted with caution as this is not a community based survey. Since the sample was drawn from a non-admitted population (healthy volunteers and ambulatory patients) visiting a tertiary care hospital, the life-time prevalence may be an overestimate. Similarly, care should be taken when generalizing these findings to urban Pakistani population. Demographics of our sample population closely match the national health survey of Pakistan data on age distribution, socioeconomic status and marital status. However our sample had more literate and more male individuals than the national average [20]. This may be partly explained by less number of females visiting the tertiary care center and their reluctance to be interviewed. Data for frequency of BDZ use was not recorded and this could be a potential limitation. We interviewed visitors and patients in ambulatory areas. Therefore some of them might have health issues which may confound our findings. We made a deliberate effort to minimize this by excluding psychiatry out-patients. Given the seriousness of this issue and in the absence of any other data, these findings are important and relevant.

Our data suggests that the lay Pakistani population is poorly educated regarding the risks and abuse potential of BDZ medications. The main source of information regarding BDZ is family and friends. Furthermore, 41% of individuals using BDZ had obtained these medications OTC, without a doctor's prescription. Even amongst those BDZ users who had received these medications through a physician, only one-third were aware of the long term abuse potential. Hence our data, in light of the easy availability of BDZ in Pakistan, suggests a grave public health risk for BDZ misuse and abuse. We and others[8] believe that there is a dire need to regulate availability of such agents over the counter. We hereby suggest a REPAIR strategy to deal with this problem. (Regulate OTC availability; physicians must Emphasize abuse potential while prescribing; Prescribe judicially and only when needed; Awareness programs for general population targeting vulnerable groups; Identification of people with dependence and their Rehabilitation).
